# Deletion of *FUNDC2* and *CMC4* on Chromosome Xq28 Is Sufficient to Cause Hypergonadotropic Hypogonadism in Men

**DOI:** 10.3389/fgene.2020.557341

**Published:** 2020-09-22

**Authors:** Xinxian Deng, He Fang, Asha Pathak, Angela M. Zou, Whitney Neufeld-Kaiser, Emily A. Malouf, Richard A. Failor, Fuki M. Hisama, Yajuan J. Liu

**Affiliations:** ^1^Department of Pathology, University of Washington, Seattle, WA, United States; ^2^The Polyclinic, Seattle, WA, United States; ^3^Department of Microbiology, Immunology and Molecular Genetics, University of California, Los Angeles, Los Angeles, CA, United States; ^4^Division of Medical Genetics, University of Washington, Seattle, WA, United States; ^5^Division of Metabolism, Endocrinology, and Nutrition, University of Washington, Seattle, WA, United States

**Keywords:** *CMC4*, *FUNDC2*, hypergonadotropic hypogonadism, Sertoli cell barrier, apoptosis, Xq28 deletion

## Abstract

**Background:**

Hypergonadotropic hypogonadism (HH) is characterized by low sex steroid levels and secondarily elevated gonadotropin levels with either congenital or acquired etiology. Genetic factors leading to HH have yet to be fully elucidated.

**Methods:**

Here, we report on genome and transcriptome data analyses from a male patient with HH and history of growth delay who has an inherited deletion of chromosome Xq28. Expression analyses were done for this patient and his unaffected family members and compared to normal controls to identify dysregulated genes due to this deletion.

**Results:**

Our patient’s Xq28 deletion is 44,806 bp and contains only two genes, *FUNDC2* and *CMC4*. Expression of both *FUNDC2* and *CMC4* are completely abolished in the patient. Gene ontology analyses of differentially expressed genes (DEGs) in the patient in comparison to controls show that significantly up-regulated genes in the patient are enriched in Sertoli cell barrier (SCB) regulation, apoptosis, inflammatory response, and gonadotropin-releasing regulation. Indeed, our patient has an elevated follicle stimulating hormone (FSH) level, which regulates Sertoli cell proliferation and spermatogenesis. In his mother and sister, who are heterozygous for this deletion, X-chromosome inactivation (XCI) is skewed toward the deleted X, suggesting a mechanism to avoid FSH dysregulation.

**Conclusion:**

Compared to the previously reported men with variable sized Xq28 deletions, our study suggests that loss of function of *FUNDC2* and *CMC4* results in dysregulation of apoptosis, inflammation, and FSH, and is sufficient to cause Xq28-associated HH.

## Introduction

Hypergonadotropic hypogonadism (HH; also termed primary hypogonadism) in men is defined by reduced production of either sperm, testosterone, or both, accompanied by elevated levels of the pituitary gonadotropins luteinizing hormone (LH) and follicle stimulating hormone (FSH; Viswanathan and Eugster, 2011; Basaria, 2014). HH can be primary, caused by constitutional genetic variants, or secondary, due to autoimmunity or exposure to chemotherapy/radiation.

The most common constitutional cause of HH in both men and women is X-chromosome abnormalities, including Turner syndrome (45,X), 47,XXY, 47,XXX, and Xq deletion (Viswanathan and Eugster, 2011), suggesting that correct dosage of specific X-linked genes determines normal gonad development. Which X-linked genes are involved in gonad development has yet to be fully elucidated. Genetic evaluation of patients with HH and X-chromosome abnormalities, especially chromosomal microdeletions spanning a few genes, may help identify candidate genes and provide new insights of the etiology of HH.

Here we report a 35-year-old man with HH, short stature, and bilateral cataracts who was identified have a 44.8 kb deletion of chromosome Xq28 encompassing *FUNDC2* (FUN14 Domain Containing 2), which encodes a mitochondrial membrane protein, and all but the shared exon 1 of *CMC4* (C-X9-C motif containing 4) and *MTCP1* (mature T cell proliferation 1). *MTCP1* encodes p13MTCP1, which is absent in normal tissue and is detected only in a rare T-cell prolymphocytic leukemia with *t*(X;14) translocations (Madani et al., 1996). *CMC4* shares a common promoter and 5′ UTR (exon 1) with *MTCP1* but has a distinct set of coding exons. *CMC4* is expressed in many tissues and encodes p8MTCP1NB, a mitochondrial membrane protein of unknown function, with highest level in fetal testis (HIPED – the Human Integrated Protein Expression Database; Madani et al., 1995; Fagerberg et al., 2014; Fishilevich et al., 2016).

To investigate the roles of *CMC4* and *FUNDC2* in gonad development and the pathways leading to HH in this patient, expression analyses including RNA-seq were done for this patient and his unaffected family members. In comparison to expression data from control males and females, we found that significantly up-regulated, but not down-regulated, genes in the patient are enriched in Sertoli cell (SC) regulation, apoptosis and inflammatory response, and gonadotropin-releasing regulation. Dysregulation of SCs, and the gonadotropin-releasing pathway in the patient is consistent with the clinical diagnosis of HH. Interestingly, it has been proposed that increased apoptosis affecting SCs and LCs is a main mechanism leading to testis dysfunction in men with 47,XXY (D’Aurora et al., 2015, 2017). In our patient’s mother and sister, who are heterozygous for the deletion, X-chromosome inactivation (XCI) is highly skewed toward the deleted X-chromosome, suggesting a mechanism in females to avoid FSH dysregulation and defects in ovarian follicular maturation in early development. Xq28 deletions have previously been reported in only eight men with syndromic forms of HH (Miskinyte et al., 2011; Lavin et al., 2016), and the minimal region of overlap with the deletion in our patient contains only exons of *CMC4* and *MTCP1*. Compared to these studies, our study suggests that loss of function of *CMC4* and *FUNDC2* leads to increased apoptosis and inflammation and damaged SC functionality, which probably causes Xq28-associated HH.

## Materials and Methods

### Protocol Approvals and Patient Consents

**FIGURE 1:**
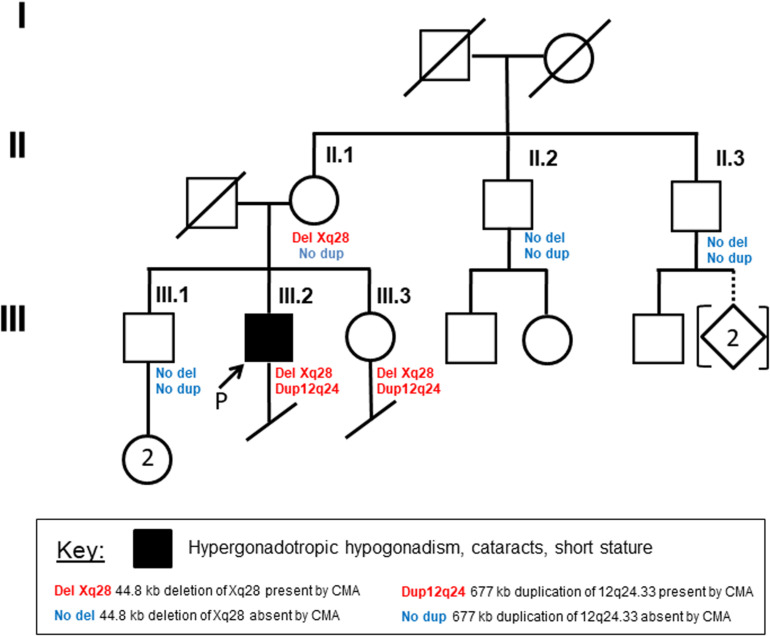
Family pedigree Circles indicate females, and squares indicate males. Diagonal lines designate deceased family members. Arrow indicates the proband. Remaining details are defined in the Key.

Written, informed consent was obtained from the patient and each of his unaffected family members, including his mother, sister, brother, and both maternal uncles (Figure 1). They were then enrolled in this study under a protocol approved by the institutional review board of University of Washington. Peripheral blood or saliva samples were obtained for DNA and RNA studies.

### Cytogenetics and Chromosomal SNP Microarray Analysis

G-banded chromosome analysis and karyotyping were done by standard methods. Interphase fluorescence *in situ* hybridization (FISH) analysis was performed on peripheral blood cells from the patient using probes for chromosomes X (DXZ1) and Y (DYZ3). Chromosomal single nucleotide polymorphism (SNP) microarray analysis of genomic DNA prepared from peripheral blood (the patient, his mother, his brother, and one maternal uncle) or from saliva (his sister, another maternal uncle) was performed using the Illumina Infinium CytoSNP-850K BeadChip v1.1. Microarray data was visualized and analyzed using Illumina BlueFuse Multi v4.4.

### PCR and Sanger Sequencing

To map the breakpoints of the patient’s deletion, DNA was isolated from peripheral blood and amplified by PCR, followed by Sanger sequencing. The PCR primers used were: TCAAAATAGACCCCATTACCAAA (forward primer); GGGGACAGCCCTTTAAGACG (reverse primer).

### mRNA-Seq

RNA-seq experiments were conducted on samples from the patient and three unaffected family members (mother, one maternal uncle, and brother) at QIAGEN Genomic Services. The library preparation was done for the starting material (100 ng) of total RNA isolated from peripheral blood using TruSeq^®^ Stranded mRNA Sample preparation kit (Illumina). The libraries size distribution was validated and quality inspected on a Bioanalyzer tapeStation (Agilent Technologies). High quality libraries are pooled for 75 nt paired-end sequencing on a NextSeq500 instrument (2 × 75 cycles) according to the manufacturer instructions (Illumina).

Reads were aligned to the human annotation reference genome GRCh38 using STAR 2.5.2b. BAM files of mapped reads were visualized by the integrative genomics viewer (IGV). 26 and 27 million (M) uniquely mapped reads (mappability: > 84%) were obtained for the patient and mother, respectively. However, only 19 M (mappability: 68%) and 8 M (mappability: 61%) uniquely mapped reads were obtained for the uncle and brother, respectively. The RNA integrity number (RIN) was very low (RIN < 3) for the samples from the uncle and brother, but RIN was > 9 for the samples from the patient and mother. Thus only RNA-seq data from the patient and his mother were used for the differential expression (DE) analysis. RNA-seq data was compared to public mRNA-seq data sets from whole blood of three males [SRR9666161 (age 33), SRR9666180 (age 32), SRR9666239 (age 40)] and three females [SRR9666175 (age 28), SRR9666197 (age 43), SRR9666240 (age 28)] age matched to the patient (RNA-seq whole blood of Dutch 500FG cohort, GSE134080). Libraries for the external controls were prepared using TruSeq mRNA Sample prep kit. The single-end read-length is 100 bp. These control data sets have a similar sequence depth and quality, with 13–17 M uniquely mapped reads (mappability: > 87%), which were reprocessed with our own data through our pipeline for normalization and comparison. Raw counts of the GENCODE genes are present in Supplementary Dataset 1, which is used for expression cutoffs for the DE analysis.

The DE analyses were done with weighted trimmed mean of M-values (TMM) normalization method and GENCODE gene annotation in the EdgeR statistical software package (Bioconductor^1^) to investigates the relative change in gene expression (i.e., normalized counts) between different samples. Six DE comparisons were conducted: (1) the patient versus three healthy male controls; (2) the patient versus three healthy female controls; (3) the mother versus three healthy male controls; (4) the mother versus three healthy female controls; (5) the patient versus the mother; and (6) male controls versus female controls. 11460 expressed genes with a median of over five raw counts per million (CPM) for the eight samples (patient, mother, three control males and three control females) were included for DE analyses. Absolute expression fold changes of two and false discovery rate (FDR) < 0.01 were set as the threshold to call genes with significant differentially expressed genes (DEGs; Supplementary Table S1–S5). DEGs were used for gene ontology (GO) enrichment analysis^2^. In addition, expression levels of genes in terms of tags per million mapped reads (TPM) and fragment per kb exon per million (FPKM) mapped are shown in Supplementary Dataset 2.

Gene ontology overrepresentation test was done by PANTHER (see footnote 2) to test whether upregulated or downregulated DE genes are enriched in GO terms (e.g., biological processes and pathways) compared to the reference of 20996 human genes. FDR < 0.05 from Fisher’s Exact test was used for the cutoff. Biological processes or pathways with enrichment fold less than one were not shown.

### Reverse Transcription PCR (RT-PCR)

Reverse transcription-PCR was done using RNA samples from peripheral blood (SuperScript III Reverse Transcriptase, Invitrogen). Specific cDNA primer pairs are (F: CACTCTTCGCATGGAGTTGA and R: AGTCCACTTGCAGCCACTCT for *F8*, spanning exons 23–25 of NM_000132.3; F: CCAGATGATGGATCAAGGCT and R: CTCTTTTGGGCCTGTATGGA for *BRCC3*, spanning exons 6–7, NM_024332.3).

### X-Chromosome Inactivation (XCI) Evaluation

X-chromosome inactivation status in the mother was evaluated by the analysis of expressed SNP reads in a few representative X-linked genes that are well expressed and subject to XCI (*ABCD1* and *SLC25A43* in this study; Supplementary Table S6; Balaton et al., 2015). The ratio of SNP read counts of one nucleotide versus total SNP reads at each SNP indicates the chance of this one to be silenced. For a gene subject to XCI, the ratio of SNP read counts of multiple SNPs at one given allele inferred from the SNP information from the patient versus total SNP reads indicates the chance of this allele to be silenced.

To further validate the results, methyl-sensitive PCR-based assay for the Androgen receptor (*AR*) locus containing the polymorphic tri-nucleotide repeat was performed for DNA from mother’s blood or from sister’s saliva sample as previously described with minor modifications (Allen et al., 1992). In brief, DNA samples were PCR amplified for the CAG repeat region (CTG from the minus strand) in the *AR* exon 1 using primers AR-F1: GCTGTGAAGGTTGCTGTTCCTCAT and AR-R1: TCCAGAATCTGTTCCAGAGCGTGC. In parallel, DNA samples were digested with the methyl-sensitive restriction enzyme HpaII followed by PCR. Sanger sequencing with AR-F1 and comparison of the amplified product from DNA with and without digestion were used to determine the presence of polymorphic repeat in test samples and the extent of XCI skewing.

## Results

### Clinical Features

A 35-year-old man with a history of HH was referred to the endocrinology clinic for ongoing management of testosterone therapy and was subsequently evaluated in the medical genetics clinic. He reported an initial diagnosis of hypogonadism about 15 years prior, during evaluation for short stature (160 cm, 1st percentile for age; mother’s height 155 cm; father’s height 188 cm). At that time, he had no dysmorphic features, and his physical exam was normal except for sparse axillary and pubic hair, small testes and penis, and prepubertal hair distribution. Testosterone replacement was initiated, and his height increased by 5 cm. In the year preceding referral to our clinic, he had discontinued testosterone therapy due to financial constraints. Without testosterone supplementation, he noticed decreased libido, lack of morning erections, low energy and motivation, difficulty concentrating, and difficulty sleeping. Review of systems was positive for depression and anxiety. He had no history of orchitis or testicular trauma, exposure to ionizing radiation or alkylating agents, liver or renal disease, abnormal bleeding, coagulopathy, developmental delay or intellectual disability, and his only previous surgery was carpal tunnel release. He reported taking no medications or using illicit drugs.

He had no family history of hypogonadism, infertility, short stature, early-onset cataracts, or abnormal development. He has a brother (height 173 cm) and sister (height 168 cm), who both went through puberty normally, and his brother has two biological daughters. He has two maternal uncles (both height 178 cm), both of whom have biological children. He is of mixed European ancestry: Scottish, English, Irish, German, and Norwegian.

Laboratory evaluation demonstrated total testosterone 15 ng/dL (250–1150 ng/dL), free testosterone 1.8 pg/dL (35.0–155.0 pg/dL), FSH 81.6 mIU/mL (0.7–10.8 mIU/mL), and LH 42.6 mIU/mL (8.6–61.8 mIU/mL), consistent with HH with > sevenfold increase of FSH. Other laboratory results included TSH 1.82 uIU/mL (0.36–3.74 uIU/mL), free T4 0.9 (0.8–1.5 ng/dL), prolactin 4.3 ng/mL (2.5–17.4 ng/mL), IGF-1 by LC/MS 173 ng/mL (53–331 ng/mL), and hematocrit 46.4% (40–52%). Fasting glucose was elevated 125 mg/dL (74–100 mg/dL), and three of six non-fasting glucose measurements were elevated (highest 135 mg/dL). HgA1C was normal twice at 5.9 and 5.8% (4.2–6.3%). Testosterone replacement was re-initiated for treatment of HH. His SNP microarray results (see below) prompted further clinical evaluation. Ophthalmologic exam revealed bilateral cataracts. Magnetic resonance imaging and angiography of the brain showed no evidence of moyamoya disease or other vascular abnormalities, and a transthoracic echocardiogram was normal. Platelet count and activated partial thromboplastin time were normal.

### Cytogenetics and Chromosomal SNP Microarray Analysis (CMA) Results

G-banded chromosome analysis and karyotyping showed a normal male karyotype (46,XY), ruling out non-mosaic 47,XXY, which is the most common cause of primary HH. Interphase fluorescence *in situ* hybridization (IFISH) analysis of peripheral blood cells using probes for chromosomes X (DXZ1) and Y (DYZ3) showed one signal each for the X- and Y-chromosomes in all 500 nuclei scored, greatly reducing the probability of mosaic XXY. CMA of peripheral blood detected two copy number variants: a minimum 677 kb duplication of chromosome 12q24.33 ([hg38] chr12:129,318,332-129,994,649), and a minimum 40.5 kb deletion of chromosome Xq28 ([hg38] chrX: 155,028,583-155,069,073). CMA showed that the patient’s mother and sister, but not his brother or two maternal uncles, have the same deletion of chromosome Xq28 (Figure 1). The 12q24.33 duplication was found only in his sister, suggesting paternal inheritance (Figure 1).

The 677 kb duplication of chromosome 12q24.33 identified in our patient contains the first four exons of *TMEM132D*, which encodes a transmembrane protein expressed in mature oligodendrocytes. Four similar duplications have been reported in the database of genomic variants (DGV), a database of copy number variants found in control populations. Two similar paternally inherited duplications have been reported in the Database of Genomic Variation and Phenotype in Humans using Ensemble Resources (DECIPHER), in patients with abnormal emotion/affect or behavior (278910) and moderate intellectual disability (280752). ClinVar lists three similar isolated duplications, all classified as of unclear significance, identified from among 29,083 people with developmental delay and intellectual disability (nssv582618, nssv3396370, and nssv579417). Our patient has no history of developmental delay, intellectual disability, congenital anomalies or dysmorphic features. We consider the 12q24.33 duplication to be an incidental finding and of uncertain significance.

We focused on the genetic and expression analyses of the Xq28 deletion in this patient with HH and his family, since Xq28 deletions have been reported in men with growth restriction and HH (Miskinyte et al., 2011; Lavin et al., 2016).

### Xq28 Deletion Breakpoint Mapping

**FIGURE 2:**
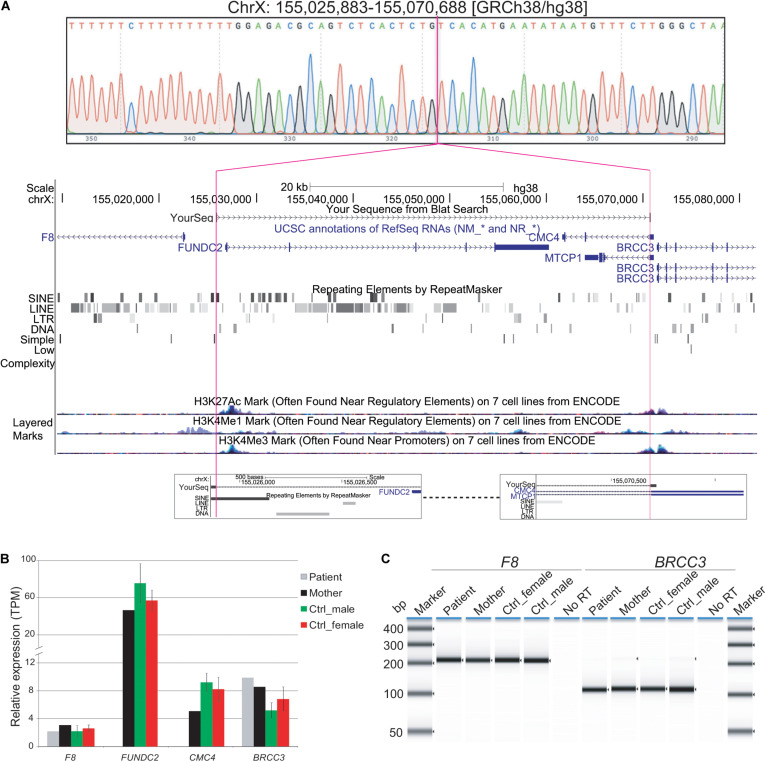
Characterization of Xq28 deletion. **(A)** 44.8 kb Xq28 deletion breakpoints (magenta lines) locate in a SINE element (breakpoint A) and intron 1 of *CMC4* and *MTCP1* (breakpoint B) as well as genes, H3K4me1/3 and H3K27Ac histone marks associated regulatory elements within and near the deletion on Xq28. **(B)** Relative expression levels of genes within and near the Xq28 deletion measured by RNA-seq. TPM, tags per million. **(C)** RT-PCR results for the expression of *F8* and *BRCC3* in peripheral blood. Error bars represent the standard deviation from the mean for three male or female controls.

Because of gaps between the probes on the microarray, the Xq28 deletion could have included a portion of exon 1 of *F8* (coagulation factor VIII, OMIM# 300841) and/or up to the first three exons of *BRCC3* (BRCA1/BRCA2-containing complex subunit 3, OMIM# 300617). Loss-of-function mutations in *F8* or in *BRCC3* cause hemophilia A or moyamoya angiopathy, respectively (Miskinyte et al., 2011; Fujita et al., 2012; Janczar et al., 2014; Lavin et al., 2016). BRCC3-containing complexes have also been proposed to be involved in regulation of SCs (Miskinyte et al., 2011). To further map the breakpoints of the patient’s deletion, DNA from peripheral blood was amplified by PCR using primers flanking the deletion, followed by Sanger sequencing. The deleted region on Xq28 was 44,806 bp (base pairs) in size ([hg19] chrX: 155,025,883-155,070,688; Figure 2A). The proximal breakpoint was in a highly repetitive sequence SINE element, 3159 bp upstream of the transcription start site (TSS) of *F8* transcript variant 1 (NM_000132). The distal breakpoint was in the first intron of *CMC4* and *MTCP1*, 732 bp upstream of the TSS of *BRCC3*. The deleted region therefore contains the entire *FUNDC2* gene plus 907 bp upstream of its TSS, and all but the shared exon 1 (5′ UTR) of *CMC4* and *MTCP1*.

### Effect of the Deletion on Local Gene Expression

Expression of *F8* or *BRCC3* could still have been impacted by the Xq28 deletion if active regulatory regions such as promoters and enhancers were located in the deleted region (Figure 2A). We tested whether expression of *BRCC3* or *F8* was affected in this patient by RT-PCR of peripheral blood. Expression levels of *F8* and *BRCC3* in the patient were similar to his mother and controls (Figure 2B).

To further identify dysregulated genes and pathways contributing to this patient’s HH, we performed transcriptome analysis by mRNA-seq of blood samples from the patient and three of his family members (mother, brother, and one maternal uncle). Raw RNA-seq read counts and gene expression levels of *FUNDC2* and *CMC4* clearly showed the absence of expression in the patient but not in family members (Supplementary Datasets 1 and 2). Note that expression of *CMC4* is not detected in the brother probably due to the poor RNA-seq quality and coverage. Since the quality of RNA-seq data for the brother and the uncle was very poor, they were excluded from further DE analysis (see Methods).

When compared to public mRNA-seq data sets from blood samples of three control males and three control females, all age matched to the patient, expression of *FUNDC2* is completely absent in the patient (Figure 1, Table 1, and Supplementary Figure S2). Since the *CMC4*/*MTCP1* region has a complex gene structure with a common promoter and 5′ exon that are not deleted in the patient (Figure 2A), we carefully examined expression of *CMC4* and *MTCP1*. There is no raw RNA-seq count for *CMC4* (GENCODE gene ENSG00000182712) after mapping using STAR (Supplementary Dataset 1), which is consistent with the complete lack of *CMC4* gene expression track from the GTEx database of any tissue. Thus *CMC4* is excluded in the DE analysis with EdgeR, which is based on the RNA-seq read counts of Genecode genes (Table 1 and Supplementary Dataset 1). However, substantial RNA-seq reads were aligned to multiple exons of *CMC4* in RNA-seq of the patient’s mother and the controls (Supplementary Figure S1), suggesting an annotation problem for the gene ENSG00000182712 for *CMC4*. In contrast, no RNA-seq read was observed on any exon in the patient, including the undeleted exon 1, probably due to degradation of the premature transcript. In support of this, TPM values calculated based on the transcript annotation were obtained for mother and the controls but not the patient (Figure 2C and Supplementary Dataset 2). *MTCP1* has substantial reads in the mother and the controls, but only on the exon 1 that is shared with *CMC4* (Supplementary Figure S1). The survey of the spliced reads covering the exon–exon conjunction clearly shows a dominant splicing event from exon 1 to the next exon of *CMC4*, but not *MTCP1* (Supplementary Figure S1B). This strongly suggests that *MTCP1* is not expressed, consistent with the previous findings of no *MTCP1* expression in normal tissues (Madani et al., 1996). We next examined expression of the deletion-flanking genes *BRCC3* and *F8*. No significantly decreased expression of the deletion-flanking genes *BRCC3* and *F8* was observed in the patient compared to male and female controls (Figure 2C and Table 1), confirming the RT-PCR results. Rather, expression of *BRCC3* was ∼twofold higher in the patient versus control males (FDR = 0.03) and females (FDR = 0.11), but very similar compared to his mother (Log2FC = 0.06; Supplementary Table S5).

**TABLE 1:** Expression changes of genes within or around chromosome Xq28 deletions.

	Patient Versus Control Males				Patient Versus Control Females			
Gene	log2_FC	log2_CPM	*P*-Value	FDR	log2_FC	log2_CPM	*P*-Value	FDR
*F8*	−0.42	2.43	0.44	0.67	−0.13	2.23	0.84	0.95
*FUNDC2*	−12.3	4.95	6E-27	6E-24	−11.8	4.36	8E-28	2E-24
*BRCC3*	1.17	3.71	0.004	0.03	1.02	3.82	0.01	0.11

	**Mother Versus Control Males**				**Mother Versus Control Females**			

*F8*	0.44	2.64	0.32	0.55	0.75	2.47	0.08	0.31
*FUNDC2*	−0.32	5.29	0.45	0.67	0.31	4.85	0.37	0.68
*BRCC3*	1.09	3.67	0.007	0.05	0.95	3.78	0.02	0.14

*Differentiation expression analysis was performed using EdgeR. FC, fold changes; CPM, counts per million; FDR, false discovery rate.*

Taken together, we found that expression of *FUNDC2* and *CMC4* is completely abolished in the patient but not in family members or normal controls, whereas expression of the deletion-flanking genes *F8* and *BRCC3* was normal and slightly increased, respectively. This is consistent with the absence of hemophilia A or moyamoya disease in the patient and also suggests a link between loss of function of *FUNDC2* and *CMC4* and the patient’s phenotype.

In contrast, the heterozygous Xq28 deletion seemed to have no significant effect on local gene expression in the patient’s mother, when compared to controls (Figure 2C and Table 1). This prompted us to examine the XCI pattern. Females inactivate one of their two X-chromosomes to achieve a similar expression level as males with one active X (Deng et al., 2014). While random XCI is present in control females, non-random or skewed XCI often occurs in females with one structurally abnormal X-chromosome. The extent of XCI skewing is correlated with the pathogenicity of the abnormality. Evaluation of expressed SNPs in the mother and the patient by RNA-seq reads in blood showed that the X-chromosome carrying the Xq28 deletion is almost completely skewed to be inactivated (> 95%) in the patient’s mother (Supplementary Table S6). XCI analysis in blood DNA using the methylation-sensitive enzyme (HpaII) assay of the *AR* polymorphic repeat locus also showed 91% of XCI skewing toward the deleted X-chromosome in the mother (Supplementary Figure S2). The patient’s sister, who is also heterozygous for the deletion, showed 82% of XCI toward the deleted X-chromosome (Supplementary Figure S2). The skewed XCI in the heterozygous mother and sister strongly suggests the importance of *FUNDC2* and/or *CMC4*. In addition, *FUNDC2* is subject to XCI (Balaton et al., 2015). Expression of the wild-type *FUNDC2* allele that is active in cells would be similar between the mother and control females, consistent with our RNA-seq analysis.

### Effect of the Deletion on Global Gene Expression

**FIGURE 3:**
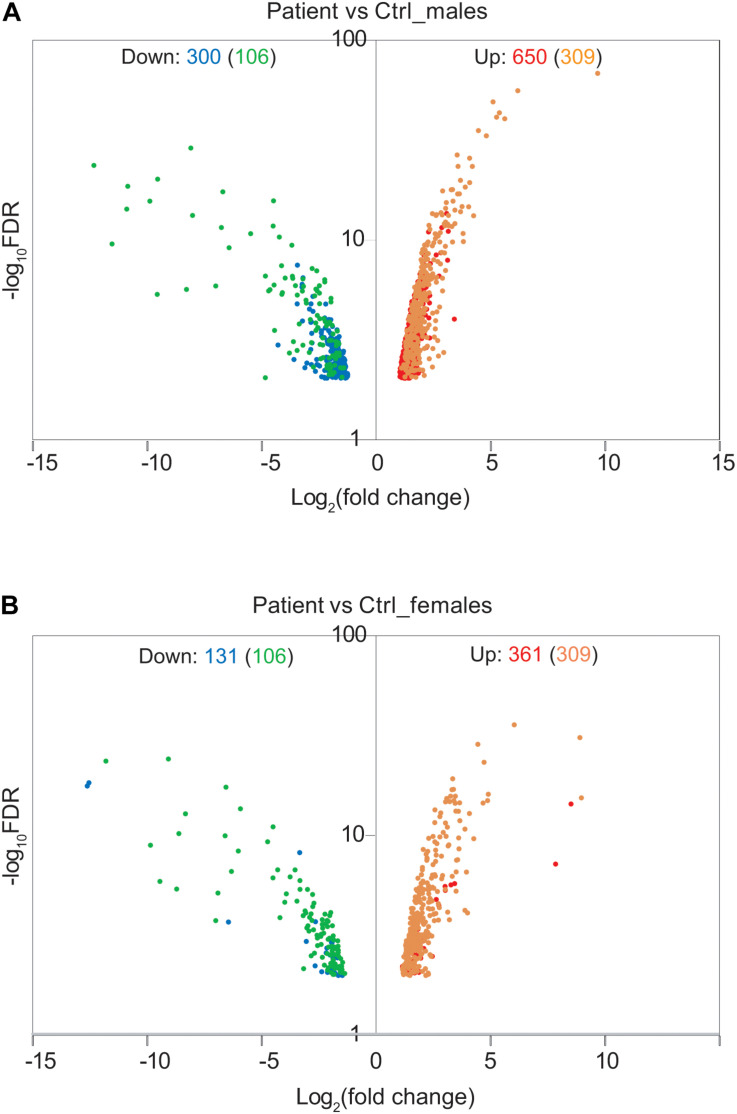
Effect of Xq28 deletion on global gene expression in the patient. **(A)** Volcano diagram of differentially expressed genes (DEGs) in the patient versus control (Ctrl) males. Horizontal axis represents expression fold change changes (log2) and vertical axis represents FDR (log10). DEGs with | log2 fold change| > 1 and FDR < 0.01 were plotted. Down- or up-regulated DEGs are shown in blue or red, respectively. Overlapped DEGs that were significantly down- or up-regulated both in the patient versus control males and in the patient versus control females are also indicated by green or yellow, respectively. The number of these overlapped DEGs is present in the parenthesis. **(B)** Volcano diagram of DEGs in the patient versus control females. The same analysis is done as in **(A)**.

**FIGURE 4:**
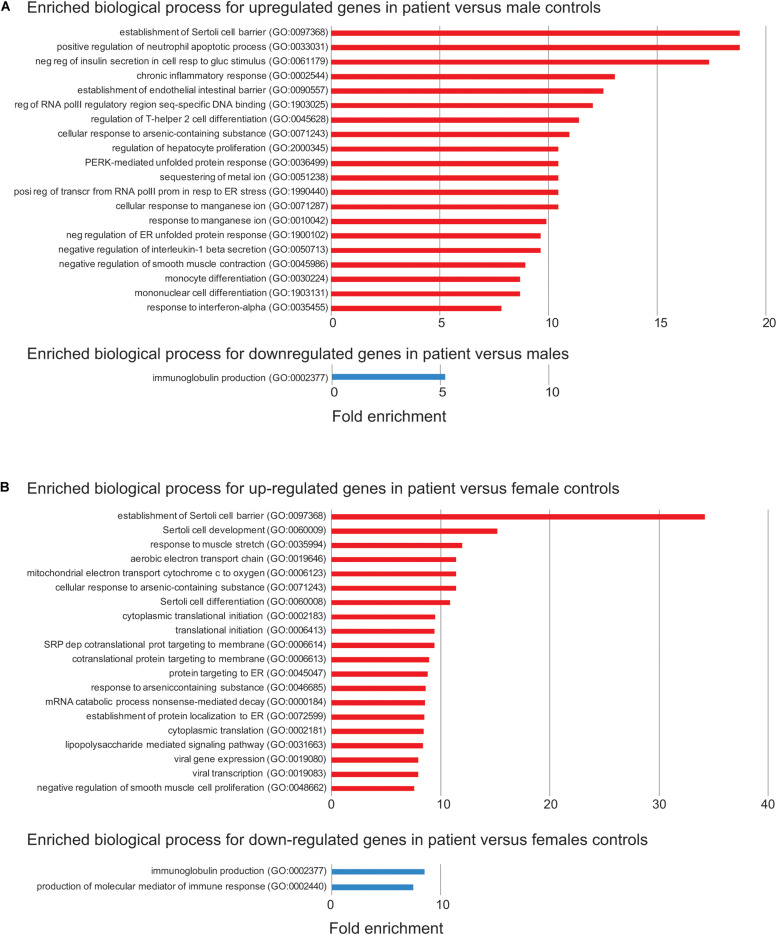
Enriched biological process for up- or down-regulated genes in the patient. **(A)** Enriched biological process from Gene Ontology (GO) analysis for up- (red) or down-regulated (blue) in the patient versus control males. The top 20 enriched biological process are shown. FDR < 0.05 was used for the cutoff. Biological process with enrichment fold less than 1 were excluded. **(B)** Enriched biological process from GO analysis for down-regulated in the patient versus control females. The same analysis is done as in **(A)**.

To address the effect of loss of function of *FUNDC2* and *CMC4* on global gene expression, we identified DEGs in the patient compared with control males or females. In the patient versus control males, 650 and 300 genes are up- or down-regulated, respectively (Figure 3A and Supplementary Table S1). GO analysis was performed for up-regulated or down-regulated DEGs separately, which has been shown to be more powerful than using all DEGs together to identify disease-associated pathways (Hong et al., 2013). Upregulated DEGs are highly enriched in biological processes relevant to the patient’s phenotype (HH and poor growth), including establishment of Sertoli cell barrier (SCB; also known as blood-testis barrier), apoptosis, cellular response to stimulus and ER stress, inflammatory response, and cell differentiation and proliferation (Figure 4A and Supplementary Table S7). Three (*ARID4A, ARID4B*, and *ICAM1*) out of five genes involved in regulation of SCB were significantly up-regulated in the patient compared to the controls (Supplementary Table S8). SCB allows Sertoli cells to control the adluminal environment in which germ cells develop by influencing the chemical composition of the luminal fluid. Damage of SCB is associated with spermatogenesis failure and testicular microenvironment deregulation (Lie et al., 2013).

Differentially expressed genes are also enriched two–sevenfold in mitochondrion function, including positive regulation of mitochondrial membrane permeability, mitochondrial ATP synthesis coupled electron transport, protein localization to mitochondrion, and mitochondrion organization (Supplementary Table S7). Overexpression of genes involved in mitochondrial function could be a way to compensate for the loss of two mitochondrial proteins encoded by *FUNDC2* and *CMC4*, respectively. No enrichment was observed for downregulated DEGs, except for minor enrichment in immunoglobulin production (Figure 4A).

In the patient versus control females, 361 and 131 genes are up- or down-regulated, respectively (Figure 3B and Supplementary Table S2). These DEGs overlap with those in the patient versus control males (Figure 3B), and the enrichment pattern is similar (Figure 4B). This again suggests that up-regulated DEGs in the patient are highly associated with the effect of loss of function of *FUNDC2* and *CMC4* and the phenotype.

Gene ontology pathway analysis of the 650 upregulated genes in the patient versus control males consistently showed enrichment of several pathways: Toll receptor signaling, apoptosis, CCKR (cholecystokinin receptor) signaling – a pathway implicated in digestion, appetite control and body weight regulation – gonadotropin-releasing hormone receptor, and inflammation mediated by chemokine and cytokine signaling (Supplementary Table S9). Almost all the top ten up-regulated genes (> 16-fold up) are involved in one or more of these pathways (Supplementary Table S1). The core up-regulated gene shared by all these pathways is *JUN*, an oncogene that encodes a protein regulating cellular proliferation and preventing apoptosis induced by TNF (tumor necrosis factor), which is also up-regulated in the patient. Interestingly, one previous analysis of the testis transcriptome in adult azoospermic patients with 47,XXY (i.e., the most common constitutional cause of HH) and control males identified a similar number of DEGs, with 656 up-regulated and 247 down-regulated genes in 47,XXY (D’Aurora et al., 2015). This study also reported that up-regulated DEGs in men with 47,XXY are enriched in biological functions and networks, including apoptosis, inflammatory response, hormone regulation and steroidogenesis, and regulation of SCB. The similarity between pathways affected in testis of men with 47,XXY and in blood of our patient with the Xq28 deletion supports the idea that disruption of a gene network involved in apoptosis, inflammation, and SCB regulation leads to HH. However, the genetic cause of HH is very different between patients with 47,XXY and our patient, who has loss of function of *FUNDC2* and *CMC4*. Only 42/650 (6.5%) and 5/300 (1.7%) of DEGs in our patient are also up- or down-regulated, respectively, in patients with 47,XXY (Supplementary Table S10). One central node gene in the top network for the up-regulated DEGs in 47,XXY is *SMAD3*, which is not affected in our patient (Supplementary Table S1). In addition, four DEGs (*ARID4A, ARID4B, ICAM1*, and *ATRX*) involved in regulation of SCB and SC development and differentiation, and found to be up-regulated in our patient, are not up-regulated in patients with 47,XXY. Interestingly, *JUN*, the core gene shared by the disrupted pathways in our patient (Supplementary Table S9), is a node gene in the top network for the up-regulated DEGs in patients with 47,XXY. Consistently, four up-regulated genes in the same pathways with *JUN* in the patient (*DUSP1, MAP4K4, TNFAIP3*, and *TUBAIA*) are also up-regulated in patients with 47,XXY, suggesting a partial overlap of targeted genes in the etiology of HH.

We also performed DE and GO analyses for the heterozygous mother, in whom the deleted X-chromosome is skewed to be almost completely inactivated. A similar set of DEGs was identified compared to controls (Supplementary Figures S4A,B, S5 and Supplementary Tables S3, S4), suggesting that deletion of *FUNDC2* and/or *CMC4* still has an effect on gene regulation in the mother. In contrast, only 16 DEGs were identified between control males and females (Supplementary Figure S3C). While *FUNDC2* is subject to XCI, *CMC4* could escape XCI and contribute to its expression levels in control females. In addition, a small portion of cells in the mother still could keep the deleted X-chromosome active since the skewing extent of XCI is ∼91%. Indeed, a relative lower expression of *CMC4* (38% down) is observed in the heterozygous mother compared to control females, which could be related to such gene dysregulation. In support of this, DE comparison of the patient and his mother only showed a small number of genes (10 up and 53 down) with expression changes (Supplementary Table S5).

Overall, these findings are relevant to the phenotype of our patient with HH in whom FSH but not LH is highly elevated, and support a role of *FUNDC2* and *CMC4* in regulation of apoptosis and inflammation.

## Discussion

We report on a man with short stature, bilateral early-onset cataracts, HH and an inherited 44.8 kb deletion of chromosome Xq28 encompassing the entirety of *FUNDC2*, exons 2–3 of *CMC4*, and exons 2–5 of *MTCP1*. Clinical evaluation excluded other known genetic and acquired causes of HH, including Klinefelter syndrome, testicular torsion, previous infection, or exposure to radiation or medications. Therefore, we evaluated him for remaining potential rare genetic causes, which led to identification of a chromosomal deletion.

This deletion has not previously been reported in the DGV, DECIPHER, or ClinVar. However, Xq28 deletions involving overlapping regions have previously been described in the medical literature, and associated phenotypically with either HH and moyamoya disease, or severe hemophilia A and moyamoya [SHAM] syndrome (Table 2 and Supplementary Table S11).

**TABLE 2:** Molecular characteristics and clinical features of male patients reported with chromosome Xq28 deletions.

Report	This Patient	Lavin et al. (2016)	Janczar et al. (2014)	Fujita et al. (2012) Patient 1	Fujita et al. (2012) Patient 2	Miskinyte et al. (2011) Family 1	Miskinyte et al. (2011) Family 2	Miskinyte et al. (2011) Family 3
Genomic coordinates [GRCh37]	chrX:154,254, 158-154,298,963	chrX:154,150, 554-154,351,604	chrX:154,210, 567-154,364,378	not specified*	not specified*	not specified*	not specified*	not specified*
Genes deleted	*FUNDC2*, *CMC4* (exons 2–3), *MTCP1* (exons 2–5)	*F8* (exons 1–14), *FUNDC2*, *CMC4*, *MTCP1*, *BRCC3*	*F8* (exons 1–6), *FUNDC2*, *CMC4*, *MTCP1*, *BRCC3*	*F8* (exons 1–22), *FUNDC2*, *CMC4*, *MTCP1*, *BRCC3*	*F8* (exons 1–22), *FUNDC2*, *CMC4*, *MTCP1*	*FUNDC2* (exons 2–5), *CMC4*, *MTCP1*, *BRCC3* (exons 1–3)	*CMC4* (exon 1), *MTCP1* (exon 1), *BRCC* (exons 1–3)	*CMC4* (exon 1), *MTCP1* (exon 1), *BRCC* (exons 1–3)
Age (years)	35	37	10	17	25	28–48	14,17	18, 26
Short stature	+	+	NR	+	–	5 of 5	2 of 2	2 of 2
Premature gray hair	–	+	NR	NR	NR	5 of 5	0 of 2	1 of 2
Eye findings	Bilateral cataracts	Unilateral proptosis	NR	NR	NR	Bilateral early-onset cataracts (4 of 5)	0 of 2	NR
Facial dysmorphism	–	NR	+	NR	NR	5 of 5	2 of 2	1 of 2
Moyamoya disease	–	– (PCA aneurysm)	+	NR	NR	4 of 5	2 of 2	2 of 2
Cardiac	Normal	NR	NR	NR	NR	DCM (3 of 5), Isolated LVE (2 of 5)	0 of 2	MI, Cardiomegaly (1 of 2)
Hemophilia A	–	+	+	+	+	0 of 5	0 of 2	0 of 2
HH	+	+	Unknown	NR	NR	5 of 5	2 of 2	NR

*not specified (deletion defined by PCR and FISH methods), HH, hypergonadotropic hypogonadism; DCM, dilated cardiomyopathy; LVE, left ventricular enlargement; MI, myocardial infarction; NR, Not reported; PCA, posterior communicating artery; TIA, transient ischemic attacks.

Miskinyte et al. (2011) described findings in seven patients from two families with syndromic moyamoya disease with HH, and a third family with syndromic moyamoya disease with no information on HH (Table 2). The critical region of overlap for syndromic moyamoya disease among these three families encompasses exon 1 of *CMC4* and *MTCP1* and the first three exons of *BRCC3* (Supplementary Table S11). *BRCC3* encodes a deubiquitinating enzyme as a member of two BRCA1 and BRISC complexes. Further experiments in this study using Zebrafish models demonstrated that deletion of *BRCC3* but not *CMC4* results in defective angiogenesis, further confirming the role of *BRCC3* in moyamoya disease. *BRCC3* was also proposed by Miskinyte et al. (2011) to play a role in HH, since *BRCC45*, a member of the BRCC3-containing complexes, is highly expressed in germ cells and SCs.

Loss-of-function variants in *F8*, which encodes coagulation factor VIII, cause hemophilia A, and microdeletions at Xq28 have been identified in patients with severe hemophilia A and moyamoya (SHAM) syndrome. Lavin et al. (2016) described a 37-year-old man with hemophilia A, who presented with left knee arthropathy and was noted to have short stature, sparse body hair, gynecomastia, prematurely gray hair, and unilateral proptosis. He was also found to have HH and a posterior communicating artery aneurysm on brain imaging. He was described as having SHAM syndrome (Table 2). Genetic evaluation revealed a 201 kb deletion of Xq28 that removed exons 1–14 of *F8* and the entirety of *FUNDC2*, *CMC4*, *MTCP1*, and *BRCC3* (Supplementary Table S11).

Janczar et al. (2014) described SHAM syndrome in a 10-year-old boy with hemophilia A who presented with focal neurologic deficits and was found on MRI (magnetic resonance imaging) to have ischemic stroke and moyamoya disease (Table 2). Genetic evaluation revealed a 150 kb deletion of Xq28 involving exons 1–6 of *F8* and the entirety of *FUNDC2*, *CMC4*, *MTCP1*, and *BRCC3* (Supplementary Table S11). Fujita et al. (2012) reported three patients with severe hemophilia with atypical intron 22 inversions and large deletions of *F8*. In two of the patients, the deletion encompassed genes other than *F8* (Supplementary Table S11). Evaluations for moyamoya disease or HH were not reported in either patient. Genetic evaluation revealed a microdeletion involving exons 1–22 of *F8* and the entirety of *FUNDC2*, *CMC4*, and *MTCP1* in both patients. The deletion extended past *BRCC3* in one of them (Supplementary Table S11).

Of the clinical features described in these previous reports of Xq28 deletions, our patient has HH, short stature, and bilateral cataracts. He has no evidence of coagulopathy or hemophilia A, cardiomyopathy, or moyamoya disease by lab tests and imaging. His phenotype is consistent with normal expression analysis of *BRCC3* and *F8* expression in blood. The critical minimal region of overlap between our patient and the previously reported 8 patients is now narrowed to exonic deletions of *CMC4* and *MTCP1*. *MTCP1* is not expressed in normal tissues, so this suggests that *CMC4* plays a role in causing HH. Consistent with this is the observation in HIPED that the level of protein encoded by *CMC4* is highest in fetal testis. Our expression analysis showed that up-regulated genes in the patient are enriched in SC regulation, gonadotropin-releasing pathway, apoptosis, and inflammatory response. This is consistent with the patient’s marked FSH elevation. It is not clear how loss of function of *CMC4* causes HH, due to very limited functional studies of this gene. One recent study using the nude mouse tumor model showed that suppression of *CMC4* (misnamed as *MTCP1* in this study, since the antibody used is for the peptide encoded by *CMC4*) by miR-126 is involved in repression of tumor growth and migration (Han et al., 2018). This suggests a role of *CMC4* in cell proliferation and mobility. How *CMC4* is associated with genes involved in SC regulation, gonadotropin-releasing pathway, apoptosis, and inflammatory response is not clear.

Although the minimal region of overlap between our patient and previously reported HH patients doesn’t include *FUNDC2*, we cannot exclude a possible role of *FUNDC2* in HH. *FUNDC2* also encodes a mitochondria protein, like *CMC4*, and regulates platelet lifespan by preventing apoptosis under hypoxia stress (Ma et al., 2019). An important paralog of *FUNDC2* is *FUNDC1*, which is highly conserved and regulates mitophagy and inflammatory response. It has been recently shown that the ortholog of *FUNDC1* in *Caenorhabditis elegans* (no *FUNDC2* in *C. elegans*) is strongly expressed in sperm and contributes to paternal mitochondria elimination in sperm (Lim et al., 2019). The most likely interacting protein for FUNDC2 is FUNDC1 as predicted by the STRING Interaction Network in the GeneCards database, suggesting functional conservation. However, it has been reported that an 18-year-old male carrying a deletion of the promoter and exon 1 of *F8* and a large deletion/insertion that removes the entire coding sequence of *FUNDC2* only showed severe hemophilia A but not HH (Sheen et al., 2007). Thus *FUNDC2* in humans could be involved in mitophagy, apoptosis/inflammation, and spermatogenesis but loss of function of *FUNDC2* alone might not cause HH. Although this result would suggest that the *CMC4* is the most likely candidate gene for HH, we can’t exclude the possibility that loss of both *FUNDC2* and *CMC4* genes cause HH.

Our patient also has bilateral cataracts. We evaluated him for cataracts because of the report of cataracts segregating with HH in four of five men with an Xq28 deletion in one family (Table 2). This is further evidence that early-onset cataracts are a variable part of the phenotype of Xq28 HH syndrome. Eye exams were not reported in several patients with a deletion of Xq28 (Table 2) so their cataract status is unknown.

The mechanism by which the Xq28 deletion increases cataract risk is not understood. Five of nine genes (*ENY2, HMGCR, PIM3, MAP4K4*, and *KLF4*) involved in negative regulation of insulin secretion in cellular response to glucose stimulus (GO:0061179; Figure 4) are upregulated in the patient. In addition, and three genes (*KLF4, RIT1*, and *VEGFA*) involved in post-embryonic camera-type eye development (GO:0031077 that is not included in the enriched biological process) are also significantly upregulated in the patient (Supplementary Table S1). These genes are all related to cell growth/apoptosis regulation. Our patient does not have diabetes, and his mild, intermittent hyperglycemia should not cause cataracts without any other diabetes complications.

## Conclusion

We describe a rare case of syndromic HH with short stature and cataracts in a 35-year-old man, in whom we identified a 44.8 kb microdeletion of chromosome Xq28 involving the entirety of *FUNDC2* and most of *CMC4* and *MTCP1*. Further genetic and expression analyses suggest a novel link between *CMC4* and *FUNDC2* and apoptosis, inflammation and FSH and SCB regulation in men, which is strongly supported by the finding that the disrupted pathways of upregulated DEGs overlap between our patient and men with 47,XXY. In addition, *CMC4* and *FUNDC2* are probably important in female-specific development, since both female family members (the mother and sister) who carry this deletion and have no clinical abnormalities have significantly skewed XCI. Our study provides novel insights into how loss of *CMC4* and *FUNDC2* contributes to dysregulation of apoptosis, inflammatory response, and HH and demonstrates the effectiveness of RNA-seq as a way to characterize gene function as part of understanding gene dosage in genetic disorders. However, precautions need to be taken in the interpretation of results from the transcriptomic analysis with a single patient against the controls, which is often the limitation of studies of rare clinical cases. Further expression studies in similar microdeletion cases of HH patients will be very helpful to confirm dysregulated genes and pathways related to the deficiency of *FUNDC2* and *CMC4*. Functional studies using transgenes to restore expression of *FUNDC2* and *CMC4* in patient cells or using genome editing to introduce the deletion in control cells will also help confirm the direct effect of the deficiency of *FUNDC2* and *CMC4* and examine their molecular mechanisms in HH.

## Web Resources

Database of Genomic Variants – http://dgv.tcag.ca

ClinVar – https://www.ncbi.nlm.nih.gov/clinvar/

DECIPHER – http://decipher.sanger.ac.uk

Gene Tissue Expression Project – http:/www.gtexportal.org

Online Mendelian Inheritance in Man – http://www.omim.org

UCSC Genome Browser – http://genome.ucsc.edu

Bioconductor – http://www.bioconductor.org/

## Data Availability Statement

The data that support the findings of this study are available on request from the corresponding author. The data are not publicly available due to privacy or ethical restrictions.

## Ethics Statement

The studies involving human participants were reviewed and approved by the institutional review board of University of Washington. The patients/participants provided their written informed consent to participate in this study. Written informed consent was obtained from the individual(s) for the publication of any potentially identifiable images or data included in this article.

## Author Contributions

XD and YL contributed to the study design, institutional review board protocol, and intellectual content of manuscript. XD, HF, AZ, WN-K, and YL analyzed and interpreted the genomic and molecular data. XD, AP, and AZ drafted the manuscript. AP, EM, RF, and FH contributed to the clinical examination of the patients in this family and interpretation of clinical data. All authors contributed to the revision and approval of the final manuscript.

## Conflict of Interest

The authors declare that the research was conducted in the absence of any commercial or financial relationships that could be construed as a potential conflict of interest.

## Footnotes

1 http://www.bioconductor.org/

2 http://geneontology.org/

## Supplementary Material

FIGURE S1 RNA-seq reads mapped at the Xq28 deletion region show that expression of *FUNDC2* and *CMC4* is completely abolished in the patient. **(A)** Mapped RNA-seq reads were binned in 100 bp windows and visualized by the integrative genomics viewer (IGV). Accumulated reads are exclusively located at exons of *FUNDC2*, *CMC4*, and *BRCC3* in the heterozygous mother and controls. Only the first exon of *MTCP1* shared with *CMC4* has substantial reads aligned. In the patient, there is a complete loss of reads at *FUNDC2* and *CMC4* but not *BRCC3*. **(B)** Individual reads visualized by IGV. Spliced reads spanning two exons were almost mapped to the exon-exon junctions for *CMC4* in the mother, suggesting that *CMC4* but not *MTCP1* is expressed. No reads are observed at *CMC4* in the patient, consistent with the loss of this gene.

FIGURE S2 Skewed X-chromosome inactivation (XCI) patterns in the heterozygous mother and sister are revealed by the methyl-sensitive PCR-based assay. **(A)** PCR results for the AR locus containing the polymorphic tri-nucleotide repeat (CTG from the minus strand as shown here). Genomic DNA from mother’s blood or from sister’s saliva sample before and after digestion with the methyl-sensitive restriction enzyme HpaII was used for PCR. **(B)** Snap shots of Sanger sequencing results for the PCR products of the AR repeat regions in the mother. Before HpaII digestion, the sample from the mother starts to show double peaks (T/A) at the position where the two alleles have different length of CTGs. This information is used to infer the number of repeats [(CTG)*_*n*_*] for each allele, which shows (CTG)_19_: (CTG)_22_ for the mother. After HpaII digestion, the allele on the inactive X which is methylated can’t be digested and becomes PCR amplified and sequenced. The ratios of the peak heights between T and A at the 1st and second positions of double peaks before and after digestion is compared to measure the ratio of XCI for each allele (e.g., similar ratios mean equal chance of XCI and increased/decreased ratios, skewed XCI). In the mother, these ratios are decreased by 10-fold after digestion, suggesting that the X-chromosome with longer repeats is preferred for digested [i.e., the other X with (CTG)_19_ is preferred for XCI]. **(C)** Snap shots of Sanger sequencing results for the PCR products of the AR repeat regions in the sister. Similar analysis was done as in **(A)**. This shows (CTG)_19_: (CTG)_24_ for the sister, suggesting the X-chromosome with (CTG)_19_ is inherited from the mother and has the Xq28 deletion. Consistently, this deleted X-chromosome is also preferred for XCI in the sister.

FIGURE S3 Effect of Xq28 deletion on global gene expression in the mother. **(A)** Volcano diagram of differentially expressed genes (DEGs) in the mother versus control (Ctrl) males. Horizontal axis represents expression fold change changes (log2) and vertical axis represents FDR (log10). DEGs with | log2 fold change| > 1 and FDR < 0.01 were plotted. Down- or up-regulated DEGs are shown in blue or red, respectively. Overlapped DEGs that were significantly down- or up-regulated both in the mother versus control males and in the mother versus control females are also indicated by green or yellow, respectively. The number of these overlapped DEGs is present in the parenthesis. **(B)** Volcano diagram of DEGs in the mother versus control females. The same analysis is done as in **(A)**. **(C)** Volcano diagram of DEGs in control males versus control females. The same analysis is done as in **(A)**.

FIGURE S4 Enriched biological process for up- or down-regulated genes in the mother. **(A)** Enriched biological process from Gene Ontology (GO) analysis for up- (red) or down-regulated (blue) in the mother versus control males. The top 20 enriched biological process are shown. FDR < 0.05 was used for the cutoff. Biological process with enrichment fold less than 1 were excluded. No any enrichment was observed for down-regulated genes. **(B)** Enriched biological process from GO analysis for down-regulated in the patient versus control females. The same analysis is done as in **(A)**.

TABLE S1 Differential expression changes between the patient and control males.

TABLE S2 Differential expression changes between the patient and control females.

TABLE S3 Differential expression changes between the mother and control males.

TABLE S4 Differential expression changes between the mother and control females.

TABLE S5 Differential expression changes between the patient and mother.

TABLE S6 Skewed X-chromosome inactivation (XCI) in the mother shown by analysis of expressed SNPs by RNA-seq in blood.

TABLE S7 Enriched biological process for upregulated differentially expressed genes (DEGs) in the patient versus control males

TABLE S8 Four upregulated differentially expressed genes (DEGs) in the patient are involved in Sertoli cell regulation in gene ontology (GO) biological process analysis.

TABLE S9 Enriched pathways for upregulated differentially expressed genes (DEGs) in the patient versus control males.

TABLE S10 A list of genes up- or down-regulated genes in the pateint versus control males and in KS (Klinefelter syndrome) patients versus control males.

DATASET 1 Raw read counts for eight RNA-seq samples.

DATASET 2 fragment per kb exon per million (FPKM) and tags per million (TPM) values for 8 RNA-seq samples.
