# Supplementary material for: Deletion of FUNDC2 and CMC4 on Chromosome Xq28 Is Sufficient to Cause Hypergonadotropic Hypogonadism in Men
Source: Front Genet. 2020 Sep 22;11:557341. doi: 10.3389/fgene.2020.557341 (PMC7537572; doi:10.3389/fgene.2020.557341)
Supplement: FIGURE S1 — RNA-seq reads mapped at the Xq28 deletion region show that expression of FUNDC2 and CMC4 is completely abolished in the patient. (A) Mapped RNA-seq reads were binned in 100 bp windows and visualized by the integrative genomics viewer (IGV). Accumulated reads are exclusively located at exons of FUNDC2, CMC4, and BRCC3 in the heterozygous mother and controls. Only the first exon of MTCP1 shared with CMC4 has substantial reads aligned. In the patient, there is a complete loss of reads at FUNDC2 and CMC4 but not BRCC3. (B) Individual reads visualized by IGV. Spliced reads spanning two exons were almost mapped to the exon-exon junctions for CMC4 in the mother, suggesting that CMC4 but not MTCP1 is expressed. No reads are observed at CMC4 in the patient, consistent with the loss of this gene. [file Data_Sheet_1.PDF]

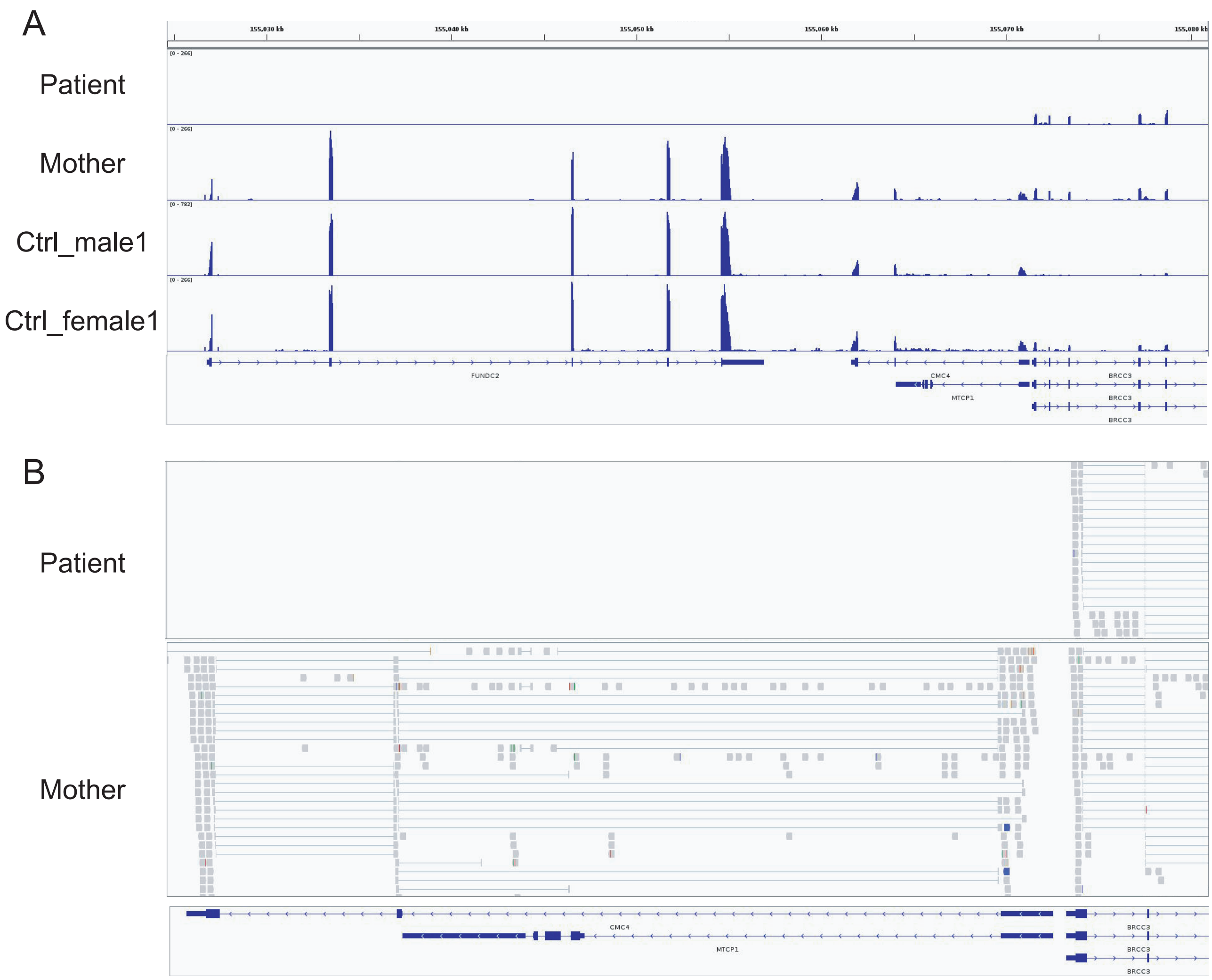

Figure S1. RNA-seq reads mapped at the Xq28 deletion region show that expression of FUNDC2 and CMC4 is completely abolished in the patient

(**A**) Mapped RNA-seq reads were binned in 100bp windows and visualized by the Integrative Genomics Viewer (IGV). Accumulated reads are exclusively located at exons of FUNDC2, CMC4 and BRCC3 in the heterozygous mother and controls. Only the first exon of MTCP1 shared with CMC4 has substantial reads aligned. In the patient, there is a complete loss of reads at FUNDC2 and CMC4 but not BRCC3. (**B**) Individual reads visualized by IGV. Spliced reads spanning two exons were almost mapped to the exon-exon junctions for CMC4 in the mother, suggesting that CMC4 but not MTCP1 is expressed. No reads are observed at CMC4 in the patient, consistent with the loss of this gene.

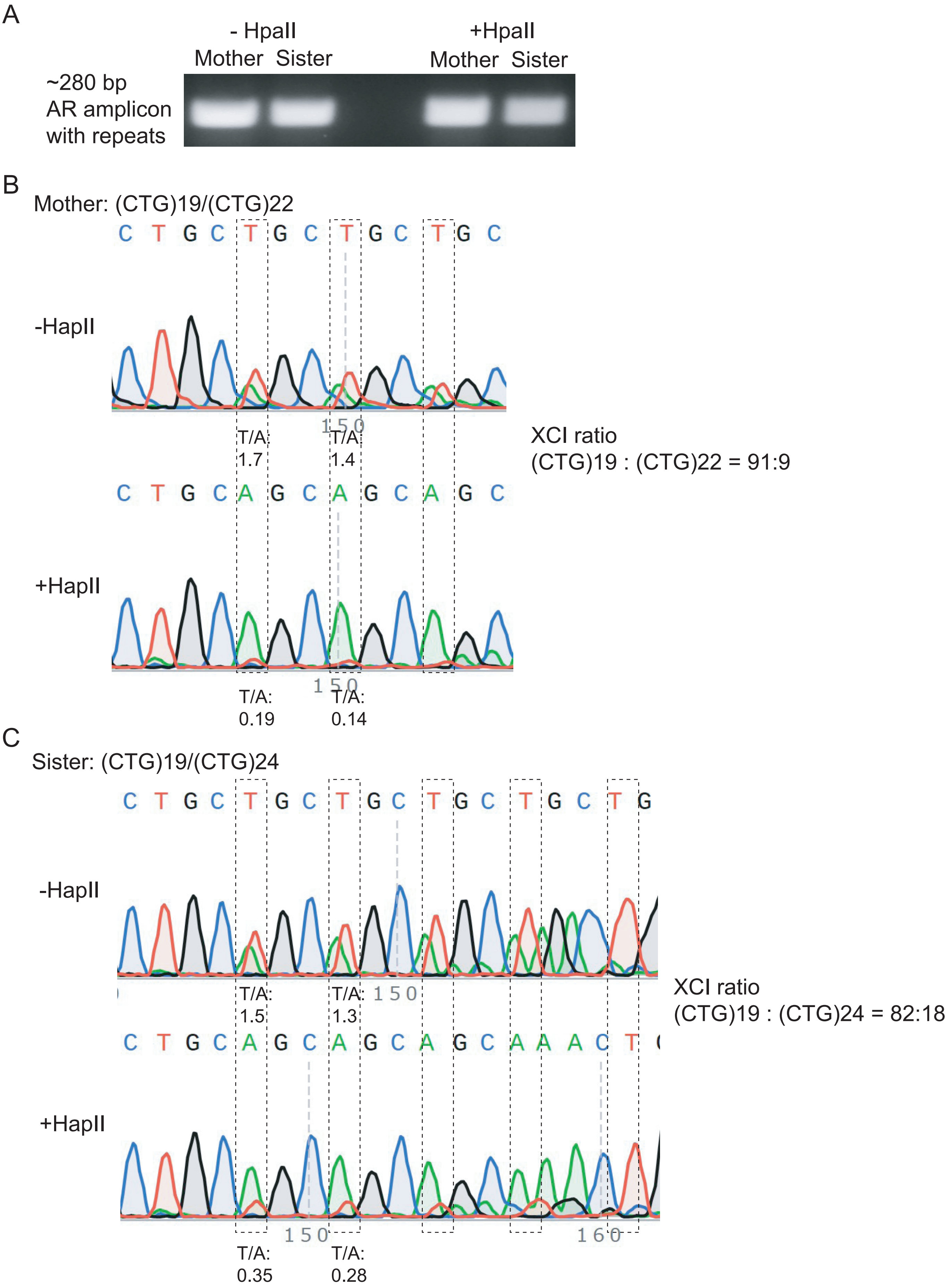
A
- HpaII
+HpaII
Mother Sister
Mother Sister
~280 bp
AR amplicon
with repeats
B
Mother: (CTG)19/(CTG)22
C T G C T G C T G C T G C
-HapII
T/A:
1.7
T/A:
1.4
XCI ratio
(CTG)19 : (CTG)22 = 91:9
C T G C A G C A G C A G C
+HapII
T/A:
0.19
T/A:
0.14
C
Sister: (CTG)19/(CTG)24
C T G C T G C T G C T G C T G C T G
-HapII
T/A:
1.5
T/A:
1.3
XCI ratio
(CTG)19 : (CTG)24 = 82:18
C T G C A G C A G C A G C A A A C T
+HapII
T/A:
0.35
T/A:
0.28

Figure S2. Skewed XCI patterns in the heterozygous mother and sister are revealed by the methyl-sensitive PCR-based as say

(A) PCR results for the AR (Androgen receptor) locus containing the polymorphic tri-nucleotide repeat (CTG from the minus strand as shown here). Genomic DNA from mother's blood or from sister's saliva sample before and after digestion with the methyl-sensitive restriction enzyme HpaII was used for PCR. **(B)** Snap shots of Sanger sequencing results for the PCR products of the AR repeat regions in the mother. Before HpaII digestion, the sample from the mother starts to show double peaks (T/A) at the position where the two alleles have different length of CTGs. This information is used to infer the number of repeats ((CTG)n) for each allele, which shows $(CTG)_{19}$: $(CTG)_{22}$ for the mother. After HpaII digestion, the allele on the inactive X which is methylated can't be digested and becomes PCR amplified and sequenced. The ratios of the peak heights between T and A at the 1st and 2nd positions of double peaks before and after digestion is compared to measure the ratio of XCI for each allele (e.g. similar ratios mean equal chance of XCI and increased/decreased ratios, skewed XCI). In the mother, these ratios are decreased by 10-fold after digestion, suggesting that the X chromosome with longer repeats is preferred for digested (i.e. the other X with $(CTG)_{19}$ is preferred for XCI). (**C**) Snap shots of Sanger sequencing results for the PCR products of the AR repeat regions in the sister. Similar analysis was done as in (**A**). This shows $(CTG)_{19}$: $(CTG)_{24}$ for the sister, suggesting the X chromosome with $(CTG)_{19}$ is inherited from the mother and has the Xq28 deletion. Consistently, this deleted X chromosome is also preferred for XCI in the sister.

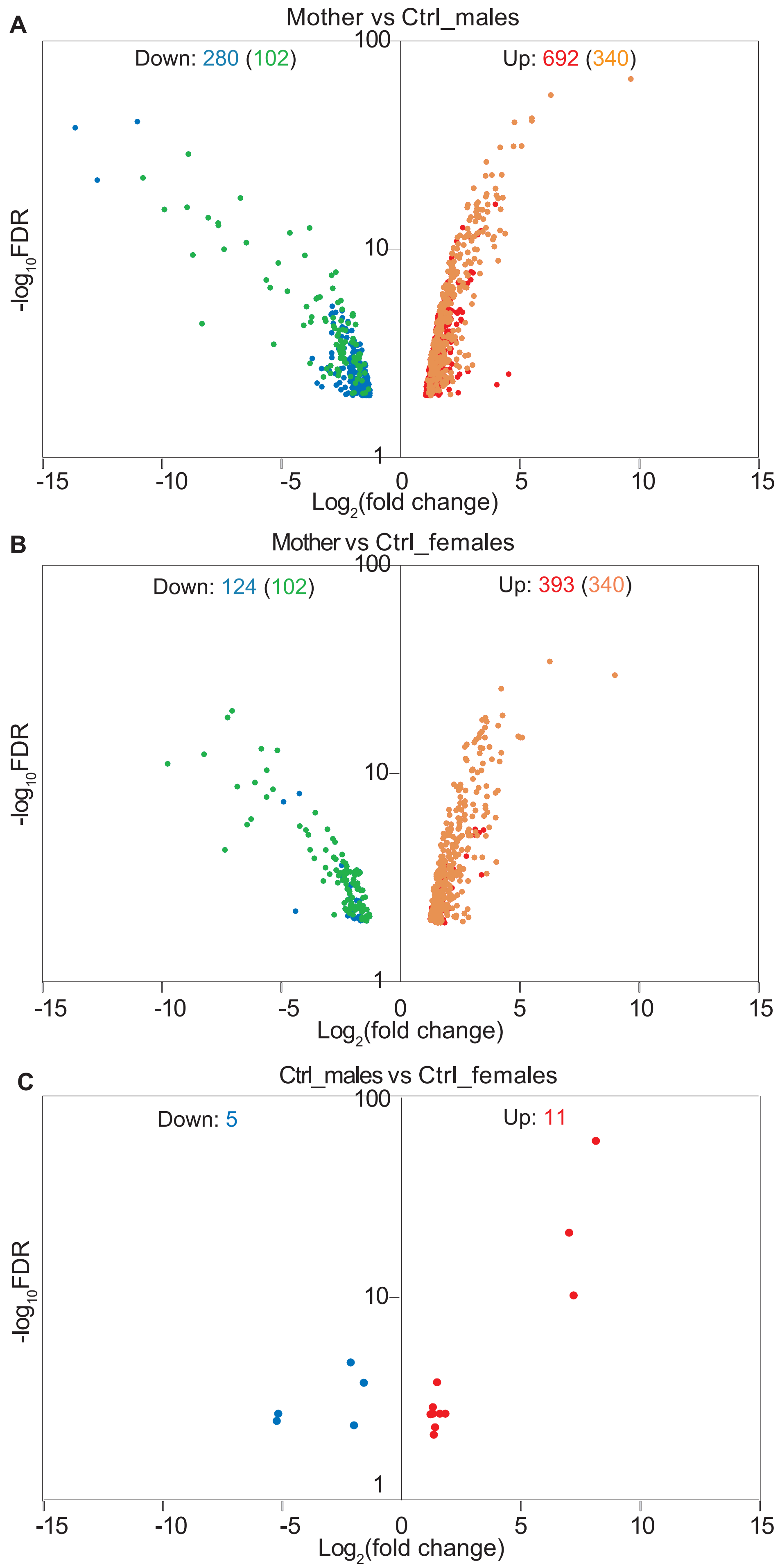

A
Mother vs Ctrl_males
Down: 280 (102)
Up: 692 (340)
100
10
1
-log10FDR
-15
-10
-5
0
5
10
15
Log2(fold change)
B
Mother vs Ctrl_females
Down: 124 (102)
Up: 393 (340)
100
10
1
-log10FDR
-15
-10
-5
0
5
10
15
Log2(fold change)
C
Ctrl_males vs Ctrl_females
Down: 5
Up: 11
100
10
1
-log10FDR
-15
-10
-5
0
5
10
15
Log2(fold change)

Figure S3. Effect of Xq28 deletion on global gene expression in the mother

(**A**) Volcano diagram of differentially expressed genes (DEGs) in the mother versus control (Ctrl) males. Horizontal axis represents expression fold change changes (log2) and vertical axis represents FDR (log10). DEGs with |log2 fold change| >1 and FDR <0.01 were plotted. Down- or up-regulated DEGs are shown in blue or red, respectively. Overlapped DEGs that were significantly down- or up-regulated both in the mother versus control males and in the mother versus control females are also indicated by green or yellow, respectively. The number of these overlapped DEGs is present in the parenthesis. (**B**) Volcano diagram of DEGs in the mother versus control females. The same analysis is done as in (**A**). (**C**) Volcano diagram of DEGs in the males versus control females. The same analysis is done as in (**A**).

A Enriched biological process for upregulated genes in mother versus male controls

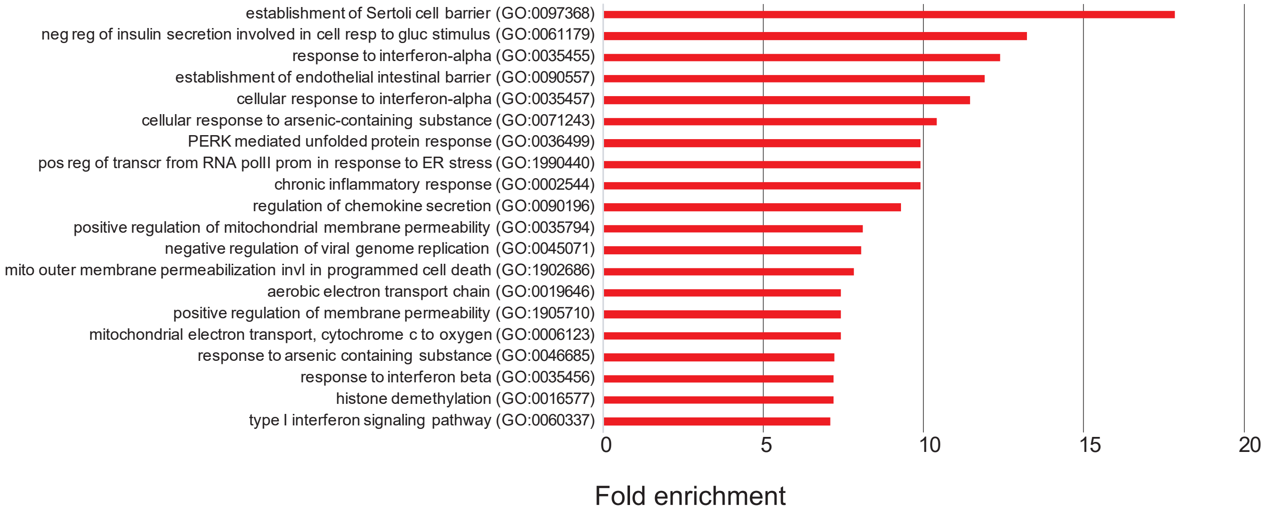

No enriched biological process for downregulated genes in mother versus males

B
Enriched biological process for up-regulated genes in mother versus female controls

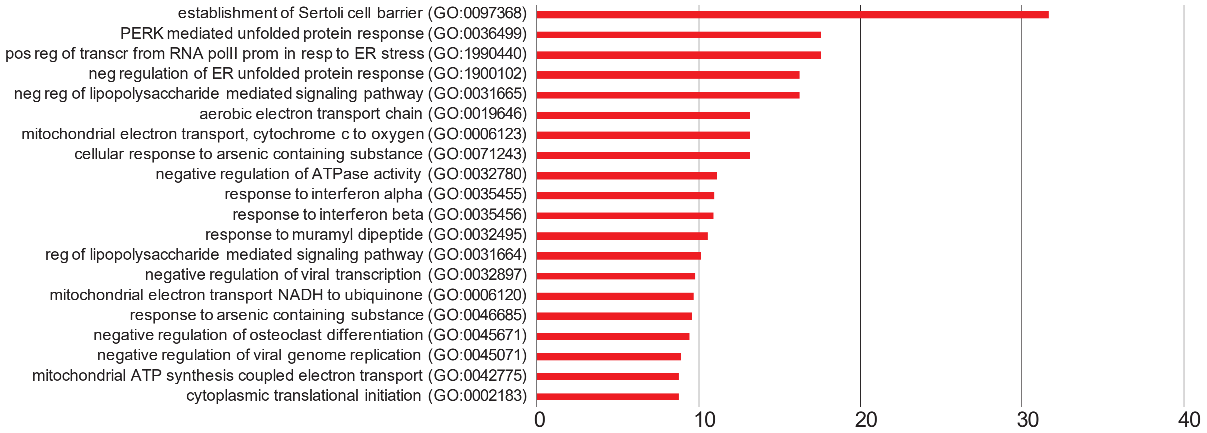

Enriched biological process for down-regulated genes in patient versus females controls

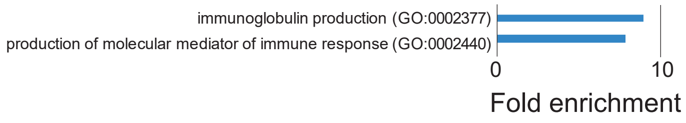

Figure S4. Enriched biological process for up- or down-regulated genes in the mother
(**A**) Enriched biological process from Gene Ontology (GO) analysis for up- or down-regulated in the mother versus control males. The top 20 enriched biological process are shown. FDR<0.05 was used for the cutoff. Biological process with enrichment fold less than 1 were excluded. No any enrichment was observed for down-regulated genes. (**B**) Enriched biological process from GO analysis for down-regulated in the patient versus control females. The same analysis is done as in (**A**).
